# Geographical accessibility in assessing bypassing behaviour for inpatient neonatal care, Bungoma County-Kenya

**DOI:** 10.1186/s12884-020-02977-x

**Published:** 2020-05-12

**Authors:** Ian A. Ocholla, Nathan O. Agutu, Paul O. Ouma, Daniel Gatungu, Felistas O. Makokha, Jesse Gitaka

**Affiliations:** 1 Department of Geomatics Engineering and Geospatial Information System, Jomo Kenyatta University of Agriculture and Technology, P.O. Box 62000-00100, Nairobi, Kenya; 2 Research and Innovation Directorate, Mount Kenya University, P.O. Box 342-01000, Thika, Kenya; 3 Bungoma County Ministry of Health, P.O. Box 14-50200, Bungoma, Kenya

**Keywords:** Bypassing behaviour, Neonatal, Geographical accessibility

## Abstract

**Background:**

Neonatal mortality rate in Kenya continues to be unacceptably high. In reducing newborn deaths, inequality in access to care and quality care have been identified as current barriers. Contributing to these barriers are the bypassing behaviour and geographical access which leads to delay in seeking newborn care. This study (i) measured geographical accessibility of inpatient newborn care, and (ii), characterized bypassing behaviour using the geographical accessibility of the inpatient newborn care seekers.

**Methods:**

Geographical accessibility to the inpatient newborn units was modelled based on travel time to the units across Bungoma County. Data was then collected from 8 inpatient newborn units and 395 mothers whose newborns were admitted in the units were interviewed. Their spatial residence locations were geo-referenced and were used against the modelled travel time to define bypassing behaviour.

**Results:**

Approximately 90% of the sick newborn population have access to nearest newborn units (< 2 h). However, 36**%** of the mothers bypassed their nearest inpatient newborn facility, with lack of diagnostic services (28%) and distrust of health personnel (37%) being the major determinants for bypassing. Approximately 75% of the care seekers preferred to use the higher tier facilities for both maternal and neonatal care in comparison to sub-county facilities which mostly were bypassed and remained underutilised.

**Conclusion:**

Our findings suggest that though majority of the population have access to care, sub-county inpatient newborn facilities have high risk of being bypassed. There is need to improve quality of care in maternal care, to reduce bypassing behaviour and improving neonatal outcome.

## Background

In 2017, approximately 7000 newborns died every day, with 36% dying on the first day, and 89% of the 2.5 million neonatal deaths occurred in-low and middle income countries [1, 2]. This unexpected high newborn deaths which constitute 47% of under-5 years mortality, has been attributed to the slow reduction in the neonatal mortality rate compared to infant and under-5 mortality reductions rate [1]**.** The World Health Organization (WHO) and the Maternal and Child Epidemiology Estimation Group estimated that 35% of all neonatal deaths in 2017 were related to preterm birth, 24% to intrapartum events such as birth asphyxia, 14% due to sepsis and meningitis and 11% were associated with congenital anomalies [3]. The three conditions (preterm birth, intrapartum-related, neonatal sepsis, and “other neonatal” conditions) contributed to 202 million disability-adjusted life years [4]. Reducing this burden requires strategies that result in timely risk identification and initiation of suitable treatment [5, 6]. This is challenging in places where most of the population does not have access to hospital-based care. Strategies to increase access to adequate services in low-income, high-burden settings are necessary as timely detection and appropriate case management can save hundreds of thousands of newborn lives [6]. Fortunately, 80% of newborn deaths are preventable through cost effective, evidence based interventions [7] such as skilled delivery, thermal care, hygienic cord care, early treatment for sepsis and promotion of breastfeeding [4, 8].

In Kenya neonatal mortality rate (NMR) remains high and stagnant over the past decade at 22 deaths per 1000 live births, compared to the national declines in infant and under-5 years mortality rates [5]. These neonatal deaths accounted for 62% (32,000 deaths) of all under five deaths in 2017 nationally [1]. Despite the government efforts on reducing neonatal mortality by 21% between 1990 and 2015 at the national level, there still exist huge disparities in access to care at the county level in preventing newborn morbidity and mortality [6].

In this era of Sustainable Development Goals (SDG), access to care and quality care especially in the high burden region of Sub-Saharan Africa (SSA) have been identified as priorities in SDG 3.2 in reducing global NMR to at least 12 deaths per 1000 live births by 2030 [9, 10]. Access to care is a multidimensional concept [11], which is based upon spatial factors (accessibility and availability) and non-spatial factors (acceptability, availability and accommodation). Our study focuses on the spatial factor of accessibility, that is, the geographical proximity of a health facility to the care seekers. There are “three delays” identified to curb global maternal mortality. The first delay is the mother’s delay in making decision to seek care, the second is the mother’s delay in reaching the facility after deciding the facility of choice and the third is the delay experienced in receiving services once after arriving at the facility [12, 13]. Modelling time travel for sick newborn population to a health facility is essential in estimating vulnerable newborn population who encounter the second delay [14].

Timely identification and management of the newborn illness is critical in their survival as their condition can rapidly deteriorate resulting in deaths [15]. It is estimated that 84% of neonatal deaths in SSA can be averted if the sick newborns are able to access care within the recommended 2 h for better management of critical newborn illnesses [16].

Besides delay in access to care, neonatal deaths are also influenced by social factors such as parental awareness of the severity of newborn illness, socio-economic status, cultural practices and means of transport to health facility [17], which often leads to delays in reaching (delay 2) and receiving quality treatment (delay 3) [14].

Evaluating these delays is essential in identifying existing gaps at care seeker individual levels and at the facility level in reduction of neonatal morbidity and mortalities [18, 19]. Poor quality of services in local facilities results in care seekers bypassing their local facilities and travelling longer distances to access better care. Bypassing behaviour is a strategy care seekers use to improve their chances of receiving quality health care and improve their health. Poor medical services [20], unavailability of health workers, lack of trust [21] in the health workers, and severity of illness [22] have been identified as causes of bypassing. Despite care seekers desire for quality care, bypassing behaviour leads to dysfunctionality in the health system [23] and delay in receiving care leads to deterioration of condition of the newborn. At the facility level, the bypassed local health facilities are underutilised [24], while the higher level facilities are over-stretched, hindering their capability to offer specialized care [23, 25]. Therefore, there is a need to understand bypassing behaviour and its mitigation management to reduce its adverse effects by objectively identifying and prioritizing care facilities which are vulnerable to bypassing.

Prior bypassing studies have often been conducted by the use of self–reported measures which are susceptible to recall bias or overestimation [24, 26]. Recently, the use of geospatial information system through the use of Euclidean distance has been implemented to overcome the limitation of self-reported in bypassing studies [24, 27]. However, this method assumes that a bypasser resides beyond a specific distance from a facility. It ignores other factors such as the terrain features, existence of geographic barriers (e.g. forests and rivers), and the mode of transport which contribute in better estimation in approximating the nearest facility to a patient residence in real world [28, 29].

In an attempt to contribute to the limited literature on how geographic accessibility can be used in assessing bypassing behaviour in a more objective way, this study, (i) estimated the geographical accessibility for care seekers to the inpatient newborn units using travel time model and (ii) characterized the bypassing extent by care seekers for the inpatient newborn units using the modelled travel time. The results of this study are critical to better decision making on reducing the second and third delays in accessing quality care for sick newborn both in terms of geographic proximity and perception of the care seekers on services rendered by the health care workers.

## Methods

### Study area

**Fig. 1:**
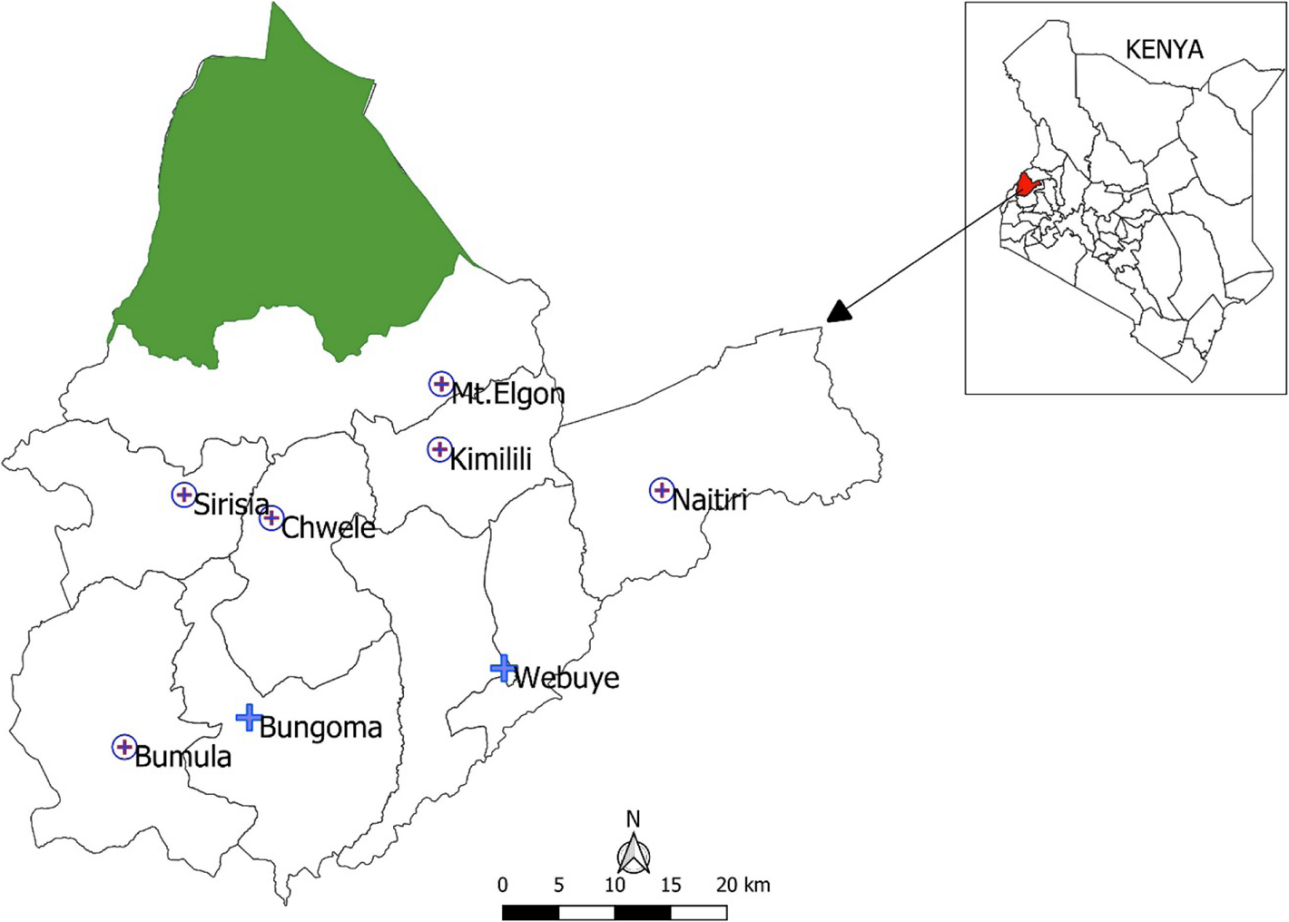
Bungoma sub counties and the eight newborn units used in the study

Bungoma County (coordinates 0.4213°N to 1.1477° N along the latitude and 34.3627° E to 35.0677° E along the longitude) is located in the western region of Kenya, bordering Uganda and covering an area of approximately 2069 km^2^. The County is administratively divided into nine sub-counties namely Bumula, Kanduyi, Sirisia, Kabuchai, Kimilili, Tongaren, Webuye East, Webuye West and Mt. Elgon. The sub-counties are divided further into forty five county assembly wards, with the county headquarters based in Bungoma town, Kanduyi sub-county (Fig. 1).

Bungoma County had an estimated population of 1.7 million in 2017 based on the 2009 National Population Census [30]. The county was purposely selected because of its high NMR of 32 per 1000 live births which is 45% above the national NMR of 22 per 1000 live births [5].

#### Health facilities

The county is served by 184 health facilities: 12 hospitals, 17 health centres, 102 dispensaries, and 52 clinics [31]. The hospitals are categorised into level IV and level V categories, with level IV facilities being sub-county hospitals which offer basic services and level V facilities representing county level facilities which offer comprehensive services. The health centres, dispensaries and clinics are categorized into lower levels in the health system hierarchy; level III, II and I respectively [32].

In an effort to reduce the high neonatal mortality in the county, eight public hospitals were selected to handle inpatient newborn incidences, the facilities included two county level facilities; Bungoma and Webuye hospitals and six sub-county level hospitals namely; Kimilili, Chwele, Bumula, Mt. Elgon, Naitiri and Sirisia (Fig. 1). In these facilities, newborn units (NBUs) were recently renovated, equipped with diagnostic equipment, and their health workers trained on newborn care [33].

### Geospatial data sources

Newborn units geographic data: The geographic coordinates of the eight selected inpatient newborn units were obtained from Google Earth [34] and existing public hospitals database [35].

#### Road network data

The road network of Bungoma county was assembled from OpenStreetMaps (OSM, 2018), then classified into primary, secondary, county and rural roads [36]. Each road segment was assigned a specific travel time based on the speed limit dependent on the road class, from a study over western Kenya [37] and Kenya National Highway Authority [38].

#### Land cover map

Land cover map was obtained from Kenya Ministry of Environment [39] at 30 m spatial resolution and was used to demarcate the land use and landcover classification. The land cover had five major classes; forestland, grassland, cropland, wetland and bare land. A digital elevation model (DEM) was obtained from the Shuttle Radar Topography Mission (SRTM) [40] at a spatial resolution of 30 m.

Lastly, a gridded surface of live births population of Kenya at 1 km spatial resolution from WorldPop database [41] was resampled to 30 m spatial resolution and used for this analysis. This gridded surface was developed from an integration of land cover data, census data and household survey data using dasymetric techniques. A detailed description of this live births layer is provided in Tatem et al. [42] and WorldPop database [41]. To estimate the inpatient newborn population, the live births population was multiplied by an estimated constant rate of burden of newborn (183/1000) who require inpatient from prior studies [43].

### Study design, sample size and procedures for bypassing data

A cross sectional inpatient newborn unit survey was conducted in the eight selected inpatient newborn facilities between May 2018 and August 2018.The purpose of the survey was to identify the care seekers hospital of admission, care seekers awareness on the nearest inpatient newborn units and the bypassing determinants.

It is approximated that there are 29,645 births annually in Bungoma County [5], however, only 21% of the newborn are born with complications requiring inpatient newborn services [33], giving a population of 6268 neonates. To estimate the sample population needed for the study, a statistical method for calculating sample population was used (Eqn1 and Eq. 2), having an allowable margin of error of 5% at a confidence interval of 95%.

1 $$ x={Z_{\raisebox{1ex}{$a$}\!\left/ \!\raisebox{-1ex}{$2$}\right.}}^2\ast p\ast \frac{1-p}{MOE^2} $$

2 $$ n=\frac{N\ast X}{\left(X+N-1\right)} $$

Where Z_a/2_ is the critical value of a normal distribution at a/2 (for a confidence level of 95% critical value is 1.96), MOE is the margin of error, p is sample proportion (50% being a default value) and N is the sample population. The sample size was estimated to be 363. All mothers whose newborn were admitted in the selected NBUs during the survey period were approached for participation until the sample size was reached.

### Study variables

The primary question in the survey was *“Is this the nearest inpatient neonatal facility to your home?”* Other independent variables used in the study were selected based on existing literature regarding their influence in bypassing behaviour on childbirth and newborn care. They included maternal age, maternal education level, occupation, and marital status, mode of transport and household assets. The name of the nearest school and village of residence of the care seeker were also required. See Additional file 1: appendix 1 for the sample questionnaire.

### Data collection

We collected the data from the care seekers (mothers) using a structured interviewer questionnaire. The questionnaire was designed in the English language but administered to the respondent either in English or translated to Kiswahili based on care seeker preference. Care seekers (mothers), in the newborn wards, whose newborns had been admitted in the inpatient NBU during this period were eligible to participate in the survey.

### Ethical review

The ethical approval was granted by the Ethical Review Committee of Mount Kenya University. Subsequent permissions were sought from the Bungoma County Ministry of Health. A written consent was obtained from the care seekers involved in the study after they were informed on the objectives of the study. For mothers who were underage (< 18 years), assent was sought from their guardians to participate in the survey. Participation was on a voluntary basis, and participants were informed of their right to withdraw their participation at any time if and when they desired.

### Generating travel time estimates using geographic accessibility model

A number of techniques have been developed to measure access to healthcare namely: gravity model [44], population provider ratio, travel time model [45] and network analysis [46]. Compared to the other methods, travel time model has been credited to be the most efficient in SSA [47, 48] and also recommended by WHO as it represents the near real world reality in accessing care [49]. Travel time model was selected as it captures and integrate corrections due to different land cover surface and terrain [28, 29, 50], it reflects most probable decision care seekers make, is intuitive and comparable across different countries [29, 51].

A geographical accessibility surface was generated using AccessMod version 5 software [52] this surface reflected the least accumulative distance or path to the nearest inpatient newborn unit [53]. In creating this surface, an initial surface impedance was created by combining data on the road network, land cover data and elevation. These surfaces were rasterized and merged into a single raster layer where travel speed was assigned to each cell at a spatial resolution of 30 m. The travel speeds for each surface and the mode of transport was assigned based on prior studies [37]. Forested areas and wetlands were assigned higher impedance values with low travel speed of 0.01 km/hr. as they acted as barriers, while low impedance values were assigned to the road networks which have high travel speed as shown in Table 1.

**Table 1:** Travel speed assigned to different land surfaces and their respective mode of transportation

Land surface(mode of transport)	Road class	Description	Speed (Km/hr.)
Primary road (Motorized)	A	Roads connecting international boundaries at specific border points.	50
	B	Connects county headquarters and other national economic centres or towns to class A road.	50
Secondary road (Motorized)	C	Link county headquarters to each other and to either class A or class B.	30
	D	Link town centers and other sub county centers to each other and links them to Class A, B or C.	30
County road (Cycling)	E	Major feeder roads that link constituency centers	11
	G	Serve to transport farm produce to the markets	11
Rural roads (walking)	L	Connects local roads to the arterial roads	11
	R	Roads in rural areas	5
	U	Unclassified rural roads	5
Dense Forest (walking)			0.01
Grassland (walking)			4.0
Cropland (walking)			4.0
Bare land (walking)			4.0
Wetland (walking)			0.01

The DEM was used to generate slope which was used to calculate different walking and cycling speeds for each degree rise based on Toblers’s equation (Eq. 3) [54].

3 $$ W=6\ast \exp \left(-3.5\  abs\left[\tan \left(\frac{S}{57.296}\right)+0.05\right]\right) $$

Where W, is the speed calculated and S is the slope in degrees.

The resulting impedance surface was used in a cost distance analysis to create an overall travel time surface to the newborn facilities. This analysis required two inputs; the selected NBU locations and the impedance surface. The cost distance tool calculated the cumulative geographic “cost”, in units of time, required to transverse from each cell in Bungoma County to the grid cell containing the selected inpatient NBU. The population of sick newborns who require inpatient care was then estimated based on aggregated inpatient newborn population at sub-county level by various travel time 30 min, 1 h and 2 h of an inpatient newborn unit using zonal statistics tool of ArcGIS (ESRI Inc.).

### Mapping to identify the closest inpatient newborn facility

The nearest school and village names were used to georeferenced the residence location of the care seekers. The georeferenced data were overlaid on the modelled geographical accessibility surface and travel time from each resident home to the nearest hospital was calculated. If the travel time to the nearest facility was less than the travel time to the facility where the newborn was admitted, the care seeker was classified as a bypasser.

### Statistical analysis

The field data were analysed using IBM SPSS Statistics Version 20 (SPSS Inc., Chicago, IL, USA). The characteristics of the mothers were analysed and compared between the bypassers and non-bypassers. Frequencies and percentages were used in analysing categorical data, while the mean and standard deviations were used for continuous data. We conducted a Principal Component Analysis (PCA) to generate wealth index quintiles of the care seekers using their household characteristics namely: source of water, type of sanitation used, source of fuel and source of lighting. Then a bivariate logistic regression analysis was carried out to assess the association between bypassing status and bypassing variables, and the results reported using odd ratios (OR), confidence interval (95% CI) and *p*-values (α = 0.05).

## Results

### Geographic accessibility to the inpatient newborn units

**Fig. 2:**
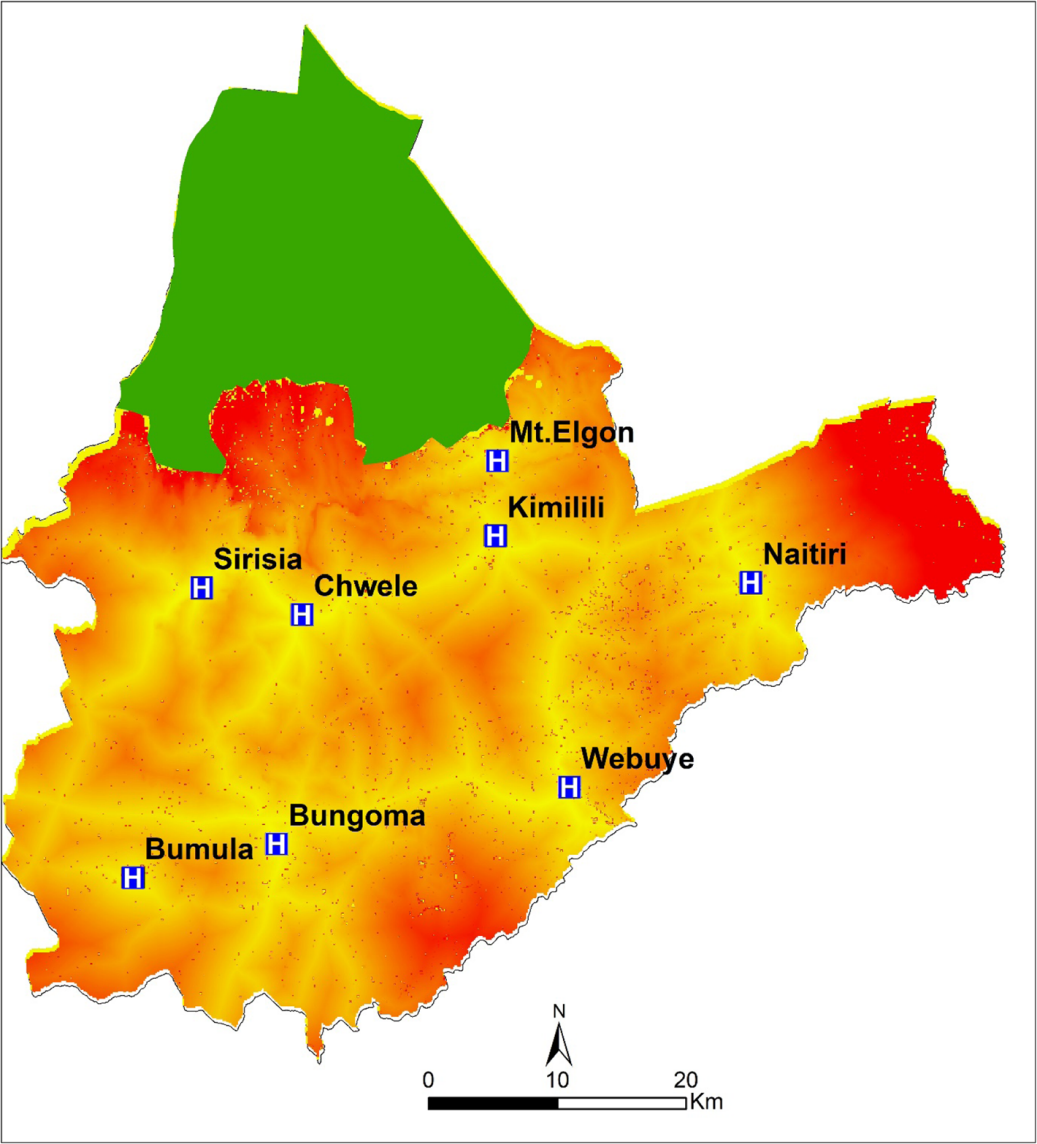
Travel time across at the county locations levels to the nearest inpatient newborn units

Using the modelled accessibility surface, travel time across the county location units was generated to show the accessibility to the inpatient newborn facility across the county (Fig. 2). The central regions of the county are well connected to primary and secondary road network and were mostly within the 2 h travel time from the nearest inpatient newborn unit. Several villages in the eastern region of the county covering Tongaren sub-county and the northern region within Mt. Elgon sub-county were beyond the recommended 2 h travel time to reach any inpatient newborn facility.

The inpatient newborn population extracted based on the modelled travel times were aggregated at the sub-county administrative level, and it was found that 90.8% of the county’s sick newborn population had access to the nearest inpatient NBU within the recommended 2 h. 7 out of 9 sub-counties had over 90% of their inpatient newborn population having access within the WHO recommended 2 h, while two sub-counties had over 20% sick newborn residing beyond 2 h to access care (Table 2). Tongaren sub-county had the highest burden of the sick and vulnerable newborn at 34% who are expected to travel more than 2 h to access the nearest inpatient newborn care, compared to Kimilili which had 0.98%. Within 60 min only two sub-counties, Kanduyi 84% and Webuye East 83%, had attained accessibility rate of above 80%, while more than 70% of inpatient newborn in Mt. Elgon sub-county could not access inpatient care.

**Table 2:** Summary of geographical access to the nearest inpatient newborn units in Bungoma County, at sub county level

Sub county	Est. inpatient newborn Population	0–30 min (%)	0–60 min (%)	0–2 h (%)
Mt. Elgon	1069	75 (7.0)	278 (26.0)	834 (78.01)
Tongaren	1122	213 (18.98)	606 (54.01)	741 (66.04)
Kanduyi	1467	675 (46.01)	1232 (83.98)	1423 (97.00)
Sirisia	687	192 (27.95)	481 (70.01)	666 (96.94)
Kabuchai	909	264 (29.04)	709 (77.99)	900 (99.00)
W. Webuye	756	159 (21.03)	514 (67.98)	703 (92.98)
E. Webuye	756	310 (41.0)	627 (82.94)	733 (96.96)
Bumula	1108	144 (12.99)	532 (48.01)	1086 (98.01)
Kimilili	824	321 (38.95)	643 (78.03)	816 (99.02)
Total	8698	2353 (27.05)	5622 (64.63)	7902 (90.84)

Values are the estimated number (and sub county percentage) of sick newborn population who require inpatient services living less than 30 min, less than 60 min or less than 2 h travel time from their nearest inpatient newborn unit

### Socio demographic characteristics of the study participants

**Fig. 3:**
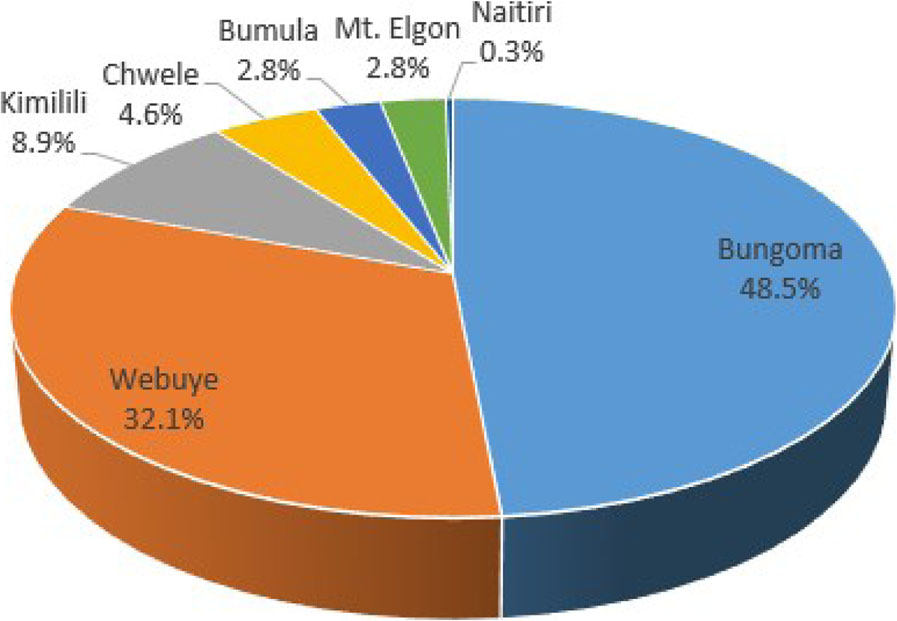
Distribution of the sick newborns admitted across the inpatinet newborn units. * Bypassed inpatient newborn units; Bgm – Bungoma, Wby- Webuye, Kml- Kimilili, Chw- Chwele, Bml- Bumula, Elg- Mt. Elgon, Sir- Sirisia, Nait- Naitiri. * Bypassing determinants; L.Drugs- Lack of drugs; H.costs- High cost; D.Equip- lack of diagnostic equipment; H.personnel- unavailability of health personnel

In the study, 395 eligible mothers of the inpatient newborns participated in the study. The mean (SD) age of the mothers was 25.04 (±4.1) with a range of 15 to 41 years. Almost half (48%) of the mother were above 25 years. County level hospitals (Bungoma and Webuye) constituted almost 80% of all inpatient newborn admissions during the study (Fig. 3). Naitiri sub-county hospital had the least number of inpatient newborns during the study period while in Sirisia sub-county facility there was no sick newborn reported requiring inpatient services.

The majority (78%) of the mothers were married, while over 56% had attained secondary education level. In terms of economic characteristics, most respondents were self-employed (35.2%) and subsistence farmers (31.9%) (Table 3). While 7 in 10 of the ill newborns were delivered in the facility of admission, three quarters (188/249 exclusion of referrals) of such cases were in the higher level facilities. Birth asphyxia was the leading cause of admission of the newborns followed by neonatal sepsis at 21 and 18% respectively as shown in Table 3 below.

**Table 3:** Background characteristics of the study participants

Characteristics	Frequency (***N*** = 395)	Percentage (%)
**Age group**		
< 20	60	15.1
20–25	144	36.5
> 25	191	48.4
**Marital status**		
Not married	87	22
Married	308	78
**Education level**		
Primary	142	36
Secondary	224	56.7
Tertiary	29	7.3
**Occupation**		
Farmers	126	31.9
Housewives	36	9.1
Self-employed	139	35.2
Unemployed	57	14.4
Employed	37	9.4
**Newborn condition**		
Birth asphyxia	84	21.3
Neonatal sepsis	73	18.5
Premature birth	73	18.5
Newborn RDS^a^	65	16.5
Low birth weight	61	15.4
Jaundice	29	7.3
Congenital disorder	1	0.3
Others	9	2.2

^a^Newborn Respiratory Distress Syndrome

### Bypassing behaviour

Among the 395 care seekers interviewed during the study, 122 (30.8%) were referral cases and 33(8.3%) were from outside study area (Bungoma county) thus were excluded from the bypassing analysis remaining with a sample population of 240. As per our definition of bypassing based on the modelled travel time, 36% (87/240) of the care seekers bypassed their nearest newborn unit. This was higher compared to the care seekers self-response on bypassing from the survey 15% (31/240). Over nine in ten (222/240) of the sick newborns were admitted at the facility where they were born.

Over three quarters of the married care seekers used their nearest newborn unit. 59% of the bypassers had secondary education compared to 8% with tertiary education. Over half (54%) of the bypassers were aged 25 year and above in comparison to 10% among care seekers age below 20 years. Similarly 75% of the bypassers were married. A higher proportion (44%) of women who belonged to the richer wealth index quintile bypassed in comparison to the poorest category (4%). Majority (78%) of the care seekers used motorcycle as the mode of transport (Table 4).

**Table 4:** Characteristic of the newborn mothers stratified by bypassing or not their nearest newborn unit

Characteristics	N = 240	Non bypasser (*n* = 153) (%)	Bypasser (*n* = 87) (%)
**Education**			
Primary	87 (36.25)	59 (38.56)	28 (32.18)
Secondary	133 (55.42)	81 (52.94)	52 (59.77)
Tertiary	20 (8.33)	13 (8.50)	7 (8.05)
**Age**			
Age < 20	30 (12.5)	20 (13.07)	10 (11.50)
20–25	94 (39.17)	64 (41.83)	30 (34.48)
> 25	116 (48.33)	69 (45.10)	47 (54.02)
**Marital status**			
Married	183 (76.25)	117 (76.47)	66 (75.86)
Unmarried	57 (23.75)	36 (23.53)	21 (24.14)
**Mode of transport**			
walking	8 (3.33)	5 (3.27)	3 (3.45)
Private car	13 (5.42)	9 (5.88)	4 (4.6)
Bus	31 (12.92)	18 (11.77)	13 (14.94)
motorcycle	188 (78.33)	121 (79.08)	67 (77.01)
**Wealth index**			
Poorest	15 (6.25)	11 (7.19)	4 (4.59)
Poorer	26 (10.83)	16 (10.46)	10 (11.49)
Middle	76 (31.67)	50 (32.68)	26 (29.89)
Richer	106 (44.17)	67 (43.79)	39 (44.83)
Richest	17 (7.08)	9 (5.88)	8 (9.20)
**Sub counties**			
Kanduyi	43 (17.92)	37 (24.18)	6 (6.90)
Kabuchai	36 (15.0)	29 (18.96)	7 (8.05)
Webuye West	30 (12.5)	19 (12.42)	11 (12.64)
Webuye East	28 (11.67)	22 (14.38)	6 (6.90)
Kimilili	28 (11.67)	12 (7.84)	16 (18.39)
Tongaren	12 (5.00)	1 (0.65)	11 (12.64)
Bumula	44 (18.33)	25 (16.34)	19 (21.84)
Sirisia	5 (2.08)	2 (1.31)	3 (3.45)
Mt. Elgon	14 (5.83)	6 (3.92)	8 (9.19)

### Which hospitals were bypassed?

Bypassing varied across the facilities with Kimilili and Bumula sub-county hospitals having highest bypassing numbers at 26.5 and 21.8% respectively. Minimal bypassers, 4% preferred other sub-county hospitals over Bungoma county referral hospital (Table 5). Bungoma and Webuye county level facilities were the most preferred by the bypassers at 41.4 and 34.5%, respectively.

**Table 5:** Bypassed newborn facilities against the preferred newborn units used by the bypassing care seekers (n = 87)

		Preferred newborn units						
		Bgm^a^	Wby^b^	Kml^c^	Chw^d^	Bml^e^	Elgon^f^	Total (%)
**Bypassed newborn facilities**	Bgm	0	0	2	0	2	0	4 (4.6)
	Wby	5	0	4	0	1	1	11 (12.6)
	Kml	5	14	0	3	0	1	23 (26.5)
	Chw	3	4	2	0	0	0	9 (10.4)
	Bml	15	6	0	0	0	0	19 (21.8)
	Elgon	0	0	3	0	0	0	3 (3.4)
	Sirisia	6	0	0	0	0	1	7 (8.1)
	Naitiri	4	6	1	0	0	0	11 (12.6)
	**Total (%)**	36 (41.4)	30 (34.5)	12 (13.8)	3 (3.4)	3 (3.4)	3 (3.4)	87

^a^Bungoma

^b^Webuye

^c^Kimilili

^d^Chwele

^e^Bumula

^f^Mt Elgon

Based on the inpatient survey, distrust for the health personnel in the bypassed facilities was cited as the main reason for bypassing (37%, *n* = 18/49). Other bypassing determinants included lack of diagnostic equipment 28% (*n* = 14/49), severity of illness 14% (*n* = 7/49), unavailability of health personnel 10% (*n* = 5/49), high cost 6% (3/49) and lack of drugs at 4.1% (2/49).

**Fig. 4:**
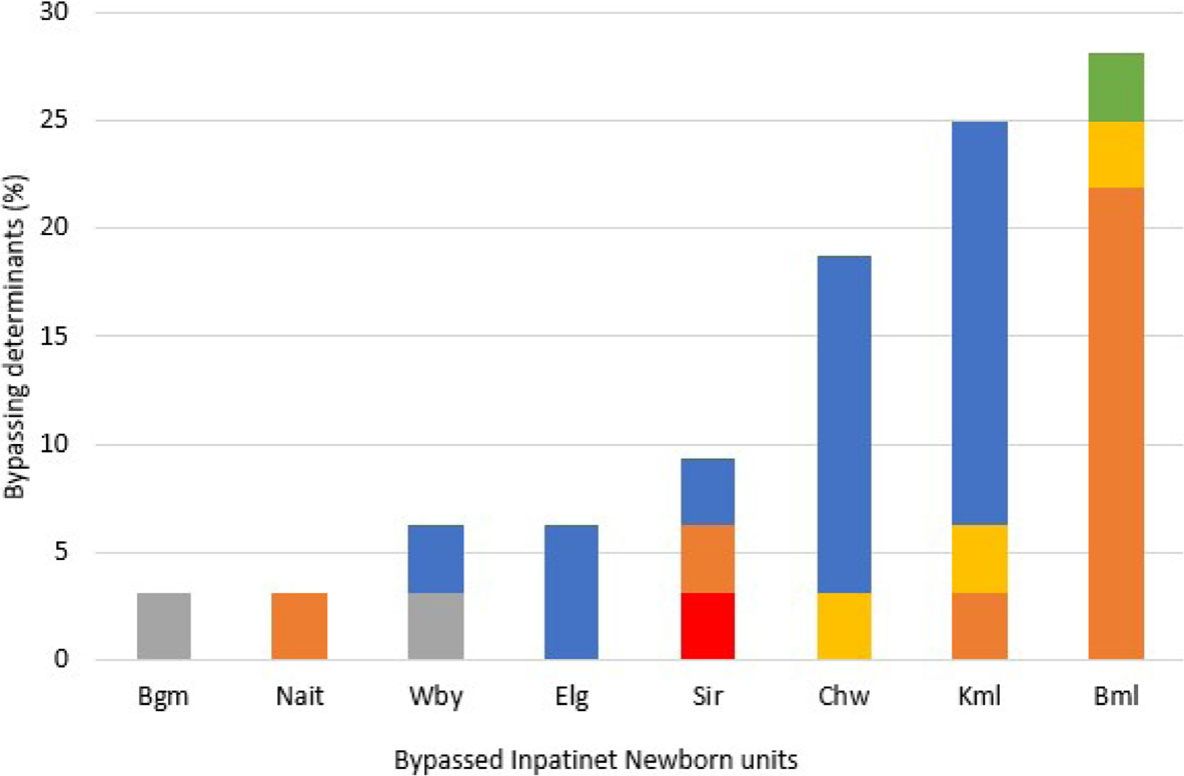
Bypassing determinants in the specific bypassed newborn units in Bungoma County based on the care seekers (*n* = 49) responses

As shown in Fig. 4 below, bypassing determinants varied across the different bypassed hospitals, with Chwele and Kimilili recording distrust in health workers as main determinants for bypassing, while in Bumula bypassers cited lack of operation of the diagnostic equipment. Bypassers of county level hospitals (Bungoma and Webuye) cited high cost in these facilities despite all facilities offering free maternal and newborn care services. The mothers are expected to pay a subsidized fee for other services offered in the facilities while on admission such as accommodation.

As shown in Fig. 4 above, unavailability of health personnel was cited only at the sub-county facilities, because these facilities are equipped with one or two personnel due to their smaller capacity compared to county level facilities which have higher capacity and more health personnel.

#### Bypassing behaviour of home delivery sick newborn

Twenty two newborns delivered at home were admitted in the newborn units. Among the twenty two, three came from neighbouring counties which were excluded from the analysis. 42% (8/19) of the mothers used their nearest newborn unit compared to (11/19) who were bypassers. 80% (9/11) of the mothers bypassed the sub-county hospitals and 72.7% (8/11) of the mother’s preferred county level facilities (Bungoma and Webuye hospitals), see Table 6. Based on the knowledge of women in this category, all the women (*n* = 19) indicated that they were using their nearest newborn units. Among this group, neonatal sepsis (31.5%) was cited as a major cause of admission in the inpatient NBU while premature birth was the lowest contributing cause at 5.2%. Based on the travel time model, three quarter of the non bypassers resided within 30 min from the nearby inpatient NBU while 72.7% of the bypassers resided beyond 30 min of nearest inpatient NBU.

**Table 6:** Analysis of ill newborns admitted at the newborns units but were delivered at home (n = 19)

Characteristic	Non bypasser (n = 8) (%)	Bypasser (*n* = 11) (%)
**Hospital level**		
Sub county	6 (75)	2 (18)
county	2 (25)	9 (82)
**Travel time**		
0–30 min	6 (75)	3 (27)
30–60 min	2 (25)	8 (73)
**Newborn illness**		
Birth asphyxia	2 (25)	3 (27.27)
Neonatal sepsis	3 (37.5)	3 (27.27)
Premature Birth	0	1 (9.09)
Respiratory disorder	1 (12.5)	3 (27.27)
Low birth weight	2 (25)	1 (9.09)

### Bivariate logistic regression analysis

Bivariate logistic regression analysis was used to assess the association between maternal characteristics and bypassing behaviour as shown in Table 7. The analysis indicated that none of the maternal characteristics was a significant predictor (*p* < 0.05) to bypassing behaviour. Care seekers with tertiary education [OR 1.134; CI 0.389–3.094] and secondary education [OR 1.352,CI; 0.769–2.406] had higher likelihood 13 and 35% respectively, of bypassing compared to those with primary education alone, while the secondary level care seekers were 22% more likely to bypass in comparison to those with tertiary education. Married women had 4% [OR 0.967; CI 0.525–1.812] less likelihood of bypassing their nearest newborn units compared to the unmarried women. Similarly, older women tended to have higher odds of bypassing compared to younger mothers in the survey.

**Table 7:** Factors influencing bypassing nearest newborn unit in Bungoma county (n = 240)

Characteristic	Estimate	*P* values	Odd ratio (95% CI)
**Education**			
Primary			1.00
Secondary)	0.302	0.297	1.352 [0.769–2.406]
Tertiary	0.126	0.808	1.134 [0.389–3.094]
**Marital status**			
Not Married			1.00
Married	−0.033	0.915	0.967 [0.525–1.812]
**Age**	0.01925	0.562	1.019 [0.954–1.088]
**Household Wealth**			
Poorest			1.00
Poorer	0.5416	0.445	1.718 [0.444–7.565]
Middle	0.3577	0.571	1.43 [0.439–5.558]
Richer	0.4705	0.446	1.60 [0.508–6.086]
Richest	0.8938	0.239	2.44 [0.571–11.779]
**Occupation**			
Unemployed			1.00
Farmer	−0.310	0.469	0.733 [0.316–1709]
Self employed	− 0.408	0.310	0.664 [0.302–1.478]
House wife	0.670	0.226	1.955 [0.666–5.933]
Employed	−0.087	0.868	0.9167 [0.322–2.555]
**Mode of transport**			
Walking			1.00
Private car	−0.300	0.751	0.740 [0.206–6.452]
Public bus or van	0.185	0.820	1.203 [0.193–3.548]
Motorcycle	−0.080	0.914	0.922 [0.333–2.462]

Bypassing was more than 2 times [OR 2.44, CI; 0.571–11.779] higher among mothers from the richest wealth index category compared to those in the poorest category. The newborn mothers who were housewives were two times [OR 1.955; CI 0.666–5.933] likely to bypass the nearest inpatient newborn unit in comparison of those who were unemployed. Likewise, newborn mothers who used public bus as means of transport had higher [OR 1.203, CI; 0.193–3.548] likelihood of bypassing compared to those who walked.

#### Spatial distribution of the care seekers

**Fig. 5:**
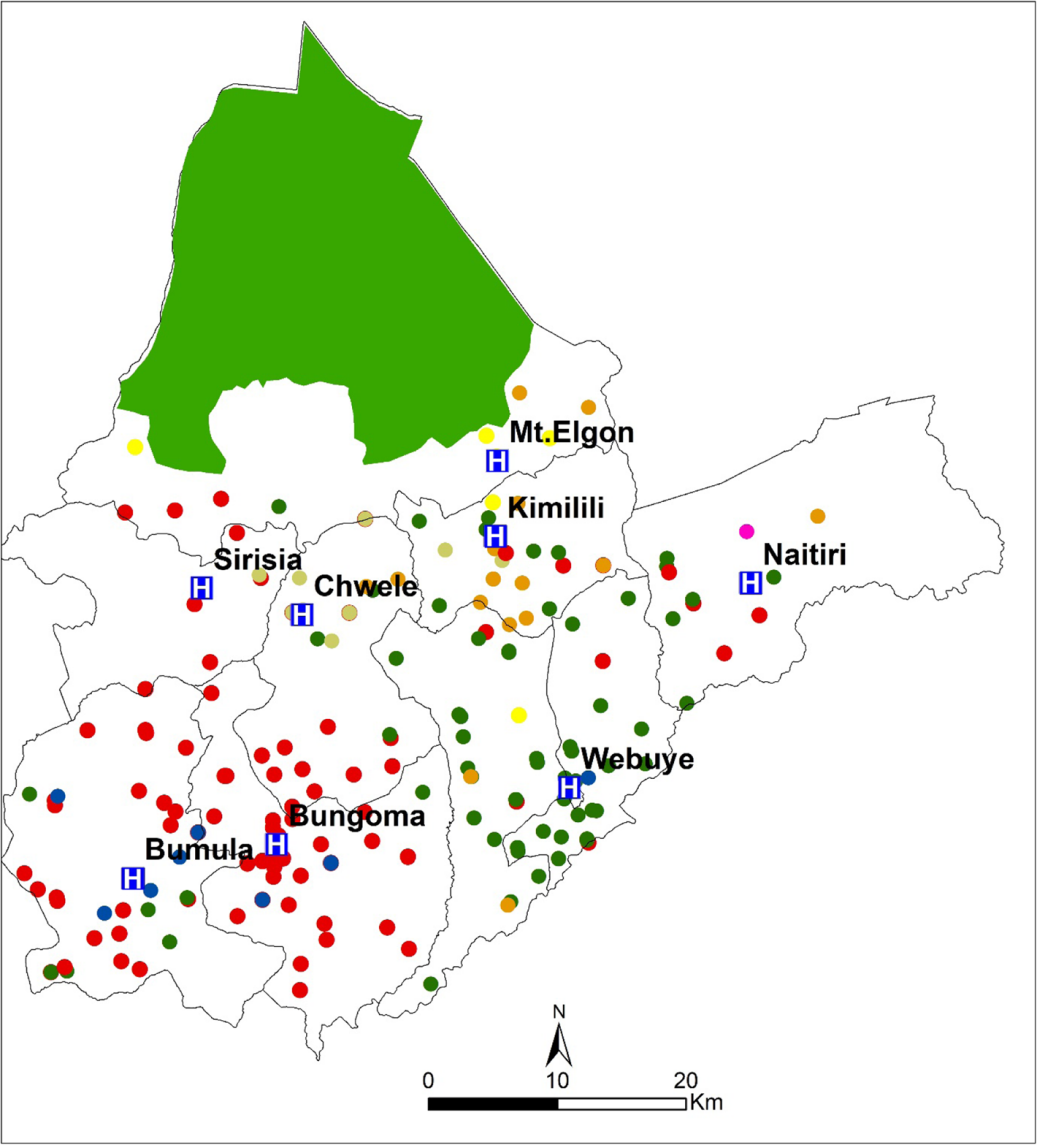
The spatial distribution of the care seekers residence and their preferred inpatient newborn facilities. *Bgm – Bungoma, Wby- Webuye, Kml- Kimilili, Chw- Chwele, Bml- Bumula, Elgon- Mt. Elgon, Sir- Sirisia, Nait- Naitiri

Care seekers probable residence location were mapped against their preferred newborn units (Fig. 5). The dots represent the care seeker residence while the dot colour represents the hospitals in which the newborn was admitted for care. It can be observed that most newborn mothers from within the locality of county level hospitals preferred to use their nearest hospitals (Bungoma and Webuye hospitals). In contrast, the pattern of facility of choice in other sub-county hospitals localities is complicated as respondent seek care from different facilities with some having to travel to county level facilities. This implies that the hierarchy of the health facility is a factor in its utilization, as most mothers sought care from county level facilities compared to the sub-county facilities.

**Fig. 6:**
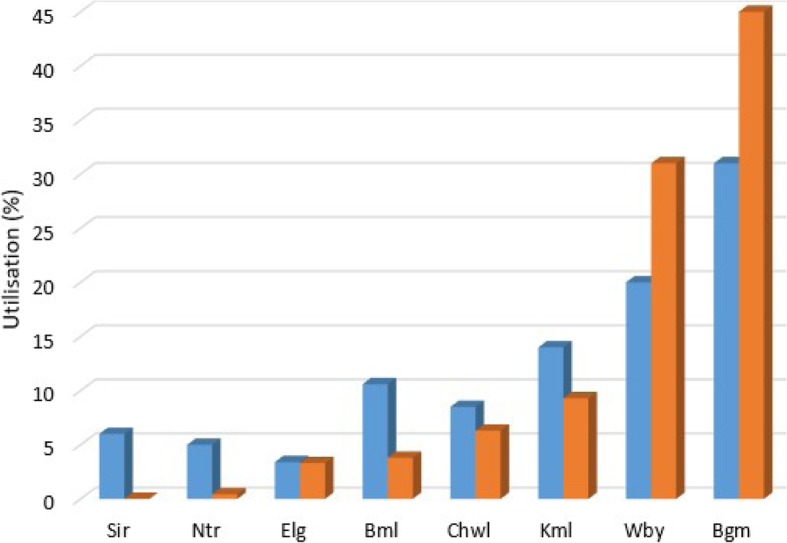
Comparison of potential and actual utilization of care seekers to the newborn units based on the inpatient data and travel time from care seekers residence to proximate newborn units (*n* = 240)

Using the respondent probable location (residence) and the modelled travel time surface, the travel time to the nearest inpatient newborn units were extracted. These extracted travel times were used to compare the utilization of the newborn units. With the potential utilization being the facilities the respondent were expected to utilize while the actual utilizations were the facilities the respondent used based on the inpatient survey. In Fig. 6 below, Bungoma and Webuye hospitals had a reduction of 14 and 11% respectively in their potential utilization compared to their actual use while all sub-county hospitals had a rise in their potential use with Bumula sub-county hospital having highest increase at 6.8% in the potential utilization. These findings indicate that the sub-county facilities are underutilised while the county level facilities may be overstretched by care seekers from all over the county.

## Discussion

This study evaluated the use of geographical accessibility in assessing bypassing behaviour of care seekers to inpatient neonatal facilities in Bungoma County. Nine in ten sick newborns have access to the nearest newborn units within the recommended less than two hours’ time [16]. Despite the high accessibility to inpatient newborn care, there are areas which are vulnerable, as sick newborns will have to travel beyond the recommended 2 h. The high accessibility within the central region of the county is attributed to a good transport network with both primary and secondary road classes that have higher speed limit resulting in lower travel time to nearest newborn facilities.

The findings show that two sub-counties recorded over 20% of sick newborns not able to reach the facilities in time (< 2 h). These data suggest that access to care is not only based on the improvement of the health facility infrastructure but also the transportation network in these areas. Poor road networks are characterised by slow speed which often results in longer travel time. The sick newborn population residing in vulnerable areas is an indication of inequality in access to care at micro level which is often obscured by the region or country level access [42].

Our results show that 36% of the care seekers bypassed their nearest inpatient newborn unit in seeking care for their ill newborns. This study rate of bypassing is similar to those reported by Kruk et al. [55] in Tanzania (42.2%) and in India (38.9%) [23], but low compared the findings in Nepal (70%) [56]. The plausible justification could be the difference in the definition of bypassing across the studies, the study population and the number of health facilities involved in the study. Unlike the prior studies which focused on birth centres [22, 23, 56] and child health care [57], our study population was specific to newborns who required inpatient neonatal care in the eight newborn units.

The main reason cited by care seekers for bypassing the nearest NBU facilities was lack of trust in the health personnel. 91% of the mothers delivered in the facilities where their newborns were admitted, suggesting that pregnant women have a preference for delivery facilities where they feel respected and they can trust the health workers [57]. Similar findings underlying this bypassing determinant were reported in rural Tanzania [55], where 69.4% of the women bypassers delivered in facility where they trusted the health workers. Lack of trust in the health providers by the mothers has been previously attributed to disrespect and abuse of women in maternity wards [58, 59]. Disrespect and abuse during childbirth in Kenya have been detailed in prior studies [58, 60], and can take the form of; physical abuse, non-consented care, lack of privacy, neglect and demands for inappropriate payments by the health facilities. Lack of respectful care has been attributed to the deficiencies in the health system such as staff shortages, inadequate training on respectful care, overburdened and unfavourable working conditions and lack of supplies and equipment [61–63]. Undignified care by the health providers consequently leads women to resort to health facilities where their dignity is upheld during childbirth.

The second reason cited for bypassing was lack of diagnostic services. Lack of surgical services in local facilities especially for maternal services such as caesarean section or high risk obstetric care often limit the utilization of the local health services. Pregnant women often prefer higher level facilities which provide comprehensive emergency services rather than local primary centres that lack these services, leading to bypassing behaviour [57, 64, 65].

Shortage of adequate drug supply in the local health facilities forces care seekers to purchase the prescribed drugs from local chemists which may be costly and also result in delays in management of critical newborn conditions [57]. This bypassing determinant has been corroborated in a study in Tanzania [21], that reported pregnant women preferred facilities with adequate drug supply during childbirth.

County level hospitals attracted not only care seekers from the nearby localities or within the same sub-county but also from other sub-counties. In addition, these facilities also had low bypassing rate, indicating preference of the care seekers to higher level facilities. These findings are consistent with previous studies in Kenya [20] and in other developing countries [22, 55]. The desire of the care seekers to attain quality care from higher tier facilities despite incurring additional transportation cost and longer travel time, is an indication of what they can sacrifice to access quality care. On the other hand, care seekers bypass their nearest sub-county facilities which are mostly faced with inefficient healthcare system [66]. However, the economically challenged population residing far away from the county level facilities may have to contend with the services offered at the local facilities. These findings provide insight for future resource allocation to these facilities and policy makers on how to strategize and attain the demand for quality care at the local facilities to minimize bypassing.

From our study, three quarters of the 91% of the mothers who delivered in the same hospital their newborn was admitted, chose to deliver in higher level facilities compared to sub-county facilities. This preference can be attributed to the perception of quality care in higher tier facilities such as; availability of supplies, availability of surgical equipment and they have a variety cadre of competent staff in case there is any complication during delivery or on the newborn to be assisted [66]. These findings underscore the importance of improving maternal care in the sub-county facilities to increase utilisation of the newborn units in the bypassed facilities.

From our findings, none of the maternal demographic characteristics was statistically significant (*p* < 0.05) to bypassing behaviour. The level of education in this study was not significantly associated with bypassing behaviour, in contrast to a study in South Africa [67] that reported higher education increased the likelihood of bypassing. However, this study findings are consistent with a study in rural Tanzania [55], in rural Chitwan Nepal [68], and in Mozambique [27]. Similarly, in other studies maternal age, marital status, [67, 69], and higher wealth index [68], have been significantly associated with bypassing behaviour. The absence of maternal characteristics being significant could be attributed to the study population size and focus on the neonatal period, which may necessarily capture mothers who have better health seeking behaviours compared to the general population. Additionally, this study was conducted in a background of health system strengthening implementation research that focused on the supply side quality improvement and demand creation [33], and thus may be reflecting impact of the ongoing study activities.

Mapping the potential and actual utilization of the inpatient newborn units based on the care seekers residence and the facility of admission validated the bypassing findings from the field data. In both, sub-county level facilities had the higher bypassing rate, indicating that the quality care in the sub-county level facilities was perceived to be lacking leading to preference for county level facilities. Also, the county level hospitals had reduction in their potential utilization, indicating that the facilities are utilised by care seekers who travelled from other hospital catchment zones to seek care in these high level facilities. This often results in overstretching of the limited resources and deviating the main role of the facilities for specialized care [23].

This study used geographic accessibility and inpatient survey to understand the bypassing patterns of care seekers for newborn care across the county. The use of modelled travel time offers a more objective measure on bypassing behaviour based on their residence location and preferred hospital accounting for geographic and infrastructure barriers. It overcomes bias or overestimation encountered in self-reported measures and limitations incurred in using straight distance for bypassing [24]. The use of geographic information system visualized the disparities that exist at sub-national level in access to care and vulnerable areas where focus should be shifted to reduce the inequalities in access.

Despite the upgrading of diagnostic equipment, improved resources of newborn units and supply of drugs majority of the facilities were not fully utilised. Our study highlights the need to focus on improving respectful maternal care both at interpersonal and impersonal levels [70], between the care provider and women especially in the sub-county facilities to reduce bypassing behaviour. Several interventions in the past studies [61, 71, 72] have been suggested and found to be successful in improving respectful care among providers namely; developing a culture of support through community dialogues; supportive supervision and management in health facilities, provision of pre and in service provider training and accountability mechanism for health providers to evaluate their values at work. These measures coupled with a functional health system characterized by availability of essential equipment and supplies and increase in human resources are some of the approaches to attain quality care that is acceptable to the care seekers at the local health facilities thereby minimizing bypassing behaviour.

The study has a number of limitations. First, the use of travel time model for geographical accessibility is imperfect. Though the model captured various factors to represent the real world landscape, the model did not consider road speed across different seasons such as rainy season, as we assumed a static dry weather landscape. The model also assumes that only the nearest facility and the optimal path were used by the mothers in accessing care. Secondly, the WorldPop live births data have inherent uncertainties of sub national data, which may affect the accuracy of the estimated proportion of inpatient newborn who have access to the newborn care units. In addition, the study was conducted in eight public sector newborn units and did not incorporate private or faith based mission facilities which offer newborn care. Therefore, the findings may not be generalised to represent the whole country, but important lessons can be drawn from the bypassing behaviour across the selected newborn units.

## Conclusion

Understanding bypassing behaviour is critical in assessing the utilization of different levels of health facilities by care seekers as expected. Our study shows that despite high geographical accessibility of reaching inpatient newborn units with the recommended 2 h, bypassing behaviour is high across the sub-county level facilities. This behaviour is seen as a strategy by care seekers to seek quality care in the higher tier facilities while underutilizing sub-county level facilities which are characterized with deficiencies in their health infrastructure.

Based on our findings, efforts to improve utilization of local newborn units should be focused on strengthening strategies that advocate for respectful and quality care in maternal care and favourable working environment for the health providers. The County Ministry of Health should also closely supervise the operations of the sub-county facilities and offer assistance where necessary to avoid underutilization of these facilities, thereby improving maternal and neonatal outcomes in the county.

## Supplementary information

**Additional file 1: Appendix 1.** Sample Questionnaire.

## Data Availability

The datasets used and / or analysed during the current study are available from the corresponding author upon request.

## Abbreviations

SSA: Sub-Saharan Africa
LMIC: Low and Middle Income Countries
WHO: World Health Organization
NMR: Neonatal Mortality Rate
NBU: Newborn Unit
DEM: Digital Elevation Model
