# Supplementary material for: Geographical accessibility in assessing bypassing behaviour for inpatient neonatal care, Bungoma County-Kenya
Source: BMC Pregnancy Childbirth. 2020 May 12;20:287. doi: 10.1186/s12884-020-02977-x (PMC7216545; doi:10.1186/s12884-020-02977-x)
Supplement: Supplementary file 1 — Additional file 1: Appendix 1. Sample Questionnaire. [file 12884_2020_2977_MOESM1_ESM.docx]

**Sample Questionnaire**

**Identification of bypassing determinants of Inpatient Newborn Unit (Questionnaire)**

Hospital name……………………………… Participant identifier……………..

Name…………………………………

Village / Location………………………………………

Nearest School……………………………………………

Age……………

Mother’s highest education: Illiterate
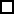
 Primary
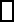
 Secondary
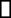
 College/University
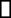


Marital status: Married
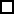
 Not Married
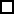
 Widow
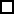
 Other (specify)……………………

(i)Household Characteristics

| What is the main source of drinking water for members of your household? *Choose one* | | | | |
| --- | --- | --- | --- | --- |
| 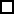 Piped water from county 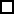 From vendor  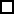 Rainwater 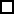 Stream/river/lake/pond  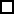 Protected spring 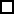 Protected well / borehole  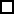 Unprotected spring 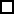 Unprotected well Other…………. | | | | |
| Do you usually do anything to the water to make it safer to drink? *Select all that apply* | | | | |
| 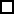 None 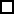 Bleach/ chlorine 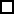 strain through a cloth  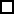 Life straw equipment 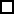 Use water filters 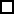 Solar disinfection 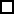 Boil 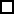 Let it stand and settle | | | | |
| What kind of toilet facility do members of your household usually use? *Select one* | | | | |
| 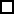Flush or pour flush toilet to piped sewer 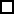 Flush to septic tank  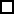 Open pit/ Pit latrine without slab 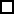 Pit latrine with slab 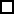 No facility / bush/ field  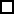Unknown | | | | |
| Do you share this toilet with other households | 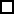 Y 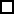 N 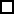 Unknown | | | |
| If Yes, including your own household, how many households use this toilet facility? | No. if < 10 ….. 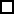 >10 households  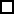 Unknown 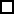 N/A | | | |
| Where is this toilet facility located? | 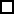 In own dwelling 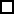 in own yard/plot  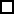 Elsewhere | | | |
| What is the MAIN cooking fuel used in this household? *Select one only* | | | | |
| 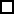 Electricity 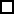 LPG/Natural gas/Biogas 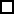 Paraffin  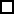 Charcoal 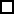 Firewood 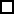 Animal Dung  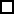 No food cooked in household 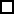 Other (specify)………………..  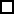 Unknown | | | | |
| What is the source of lighting in your household? | | | | |
| 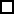 Electricity 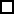 Lamps (paraffin) 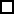 Solar power 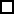 Firewood  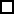 Other (specify)……………….. | | | | |
| Does this household own any livestock, herds, other farm animals or poultry | | 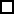 Y | 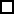 N | 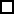 Unknown |
| If yes, how many of the following animals does this household own? | | | | |
| Cows/bulls ….. Sheep………..  Horses/Donkey/Mules…… Goats……….  Chickens or Ducks ……… Others……….. Number……… 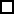 N/A | | | | |
| What is your occupation? | | | | |
| 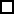 Farming 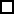 Business 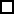 Job-Employed 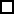 Housewife 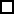Not employed 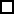 Student 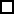 Other (specify)………………………….. | | | | |

(ii) Hospital admission

| Did you deliver your baby in this Hospital | | | 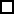 Y | | | | 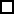 N | | | |
| --- | --- | --- | --- | --- | --- | --- | --- | --- | --- | --- |
| If N, Where did you deliver your baby? | | |  | | | | | | | |
| Were you referred here | | | 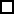 Y | | | | 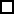 N | | | |
| **REFERRAL CASE (ONLY-from Health facilities)** | | | | | | | | | | |
| If YES, from which hospital/ facility | | |  | | | | | | | |
| Which Kind of hospital is it? | | | 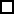 Public | | | 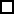 Private | | | | 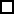 Faith based |
| Why were you referred? | | | | | | | | | | |
| 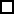 Lack of Drugs 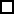 Lack of diagnostic services (equipment)  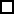 High cost for treatment 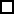 Unavailability of health workers  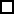 Severity of the illness 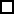 Poor services  Other (specify)…………………… | | | | | | | | | | |
| Which mode of transport did you use to come to this facility | | | | | | | | | | |
| 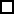 Walking 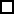 Private vehicle 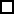 Ambulance 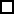 Matatu  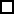 Bicycle 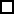 Bus 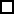 Motorcycle  Others (specify)………………………………….  If multiple modes, indicate all. | | | | | | | | | | |
| **IF YOU WERE NOT REFERRED** | | | | | | | | | | |
| Is this the nearest newborn unit from your household | | | | | 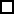 Y | | | | 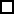 N | |
| If N, which one is the nearest? | |  | | | | | | | | |
| Which kind of hospital is nearest to your home? | 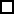 Public | | | 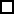 Private | | | | 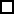 Faith based | | |
| Why did you bypass the nearby facility? | | | | | | | | | | |
| 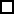 Lack of Drugs 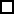 Long duration in seeking treatment 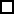 Lack of diagnostic services  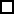 High cost for treatment 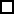 Unavailability of health workers  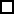 Lack of trust in the health workers 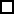 Severity of the illness 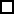 Not open  Other (specify)…………………… | | | | | | | | | | |
| Which mode of transport did you use to come to this facility | | | | | | | | | | |
| 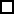 Walking 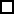 Private vehicle 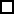 Ambulance 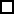 Matatu  Bicycle Bus Motorcycle  Others (specify)………………………………….  If multiple modes, indicate all. | | | | | | | | | | |
| Estimate the time taken (in minutes) to reach this hospital from home | | |  | | | | | | | |
| How long has your baby stayed in the NBU? | | |  | | | | | | | |
| What are the reasons for admission? | | | | | | | | | | |
| Birth Asphyxia Neonatal Sepsis Jaundice  Premature birth Newborn RDS Low Birth weight  Congenital anomalies Preterm Unknown  Other (specify)…………………………………….. | | | | | | | | | | |
| Have you noticed any improvement in your baby? | | |  | | | | | | | |
| How do you find the services of this Newborn unit? | | | | | | | | | | |
| Availability of Drugs Short duration in seeking treatment  Availability of diagnostic services  Affordable Cost for treatment Availability of health workers  Trust in the health workers  Other (specify)…………………… | | | | | | | | | | |
| What do u think should be improved on within the NBU? | | | | | | | | | | |
| Availability of Drugs Availability of more diagnostic equipment’s  Affordable Cost for treatment Availability of more health workers  Other (specify)…………………… | | | | | | | | | | |
